# Selective Growth of Bulk‐Like Perovskite in Plasmonic Nanoholes for Enhanced Two‐Photon‐Excited Emission

**DOI:** 10.1002/adma.73835

**Published:** 2026-07-09

**Authors:** Jung‐Jae Do, Bryan K. Chantigian, Daniel Upcraft, Wonwoo Lee, Ho‐Sung Shin, Nathan C. Lindquist, Jae Woong Jung, Bharat Jalan, Sang‐Hyun Oh

**Affiliations:** ^1^ Department of Chemical Engineering and Materials Science University of Minnesota Minneapolis Minnesota USA; ^2^ Department of Electrical and Computer Engineering University of Minnesota Minneapolis Minnesota USA; ^3^ Department of Physics and Engineering Bethel University St Paul Minnesota USA; ^4^ Department of Materials Science and Engineering College of Engineering Kyung Hee University Yongin‐si Gyeonggi‐do Republic of Korea; ^5^ Integrated Education Institute for Frontier Materials (BK21 Four) Kyung Hee University Yongin‐si Gyeonggi‐do Republic of Korea

**Keywords:** halide perovskite (CsPbBr_3_), in situ crystallization, nanohole metasurface, peel‐off technique, plasmonics, two‐photon absorption

## Abstract

Plasmon‐enhanced nonlinear optical phenomena in hybrid perovskite‐plasmonic systems present significant opportunities for photodetection, bioimaging, and quantum light sources. However, achieving uniform growth of luminescent materials within confined plasmonic hotspots remains challenging, as conventional surface deposition or infiltration approaches cannot fill nanocavities effectively. To resolve this, we present a vacuum‐assisted capillary infiltration (VACI) strategy for delivering precursor solutions into gold (Au) nanohole (NH) arrays, enabling in situ crystallization within plasmonic cavities. This work demonstrates selective bulk‐like cesium lead bromide (CsPbBr_3_) growth inside NHs through precise infiltration and controlled crystallization. By integrating the capillary effect with vacuum pressure, this approach ensures uniform filling beyond conventional methods. The successful integration of CsPbBr_3_ was confirmed through peel‐off techniques and structural/chemical analyses. Electromagnetic simulations predict an average field enhancement of |*E/E_0_* | ≈2.80 at 800 nm, consistent with an experimentally observed ∼40‐fold increase in two‐photon absorption (TPA)‐induced green emission (*λ* ≈526 nm). Unlike prior TPA systems employing quantum dots, rare‐earth doped crystals, or organic chromophores, this approach integrates bulk‐like perovskite directly into plasmonic NH cavities. This establishes a general framework for material‐structure integration in plasmonics and nanophotonics, and a foundation for infrared photodetection, high‐resolution nonlinear imaging, and quantum light sources.

## Graphical abstract

Vacuum‐assisted capillary infiltration delivers perovskite precursors deep into gold nanohole plasmonic hotspots, enabling uniform in situ crystallization of bulk‐like CsPbBr_3_ within confined cavities. Verified by peel‐off and structural analyses, the hybrid platform shows simulated |E/E₀| ≈2.80 at 800 nm and ∼40‐fold two‐photon absorption‐enhanced green emission, advancing infrared detection, nonlinear bioimaging, and quantum light sources.

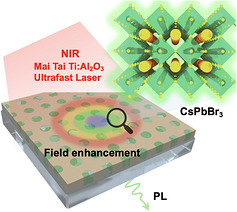

## 1 Introduction

Metal halide perovskites (CsPbX_3_, X = Cl, Br, I) have emerged as next‐generation optoelectronic materials owing to their exceptional properties, including high absorption coefficients, outstanding photoluminescence quantum yields (PLQYs), long carrier diffusion lengths, and low‐cost, large‐area processability through solution‐based fabrication techniques [1, 2, 3, 4, 5]. In particular, CsPbBr_3_, with a direct bandgap of ≈2.4 eV and efficient green emission, is a promising candidate for applications such as light‐emitting diodes (LEDs) [6, 7], photovoltaics [8], lasers [9], and photocatalytic reactors [10]. Despite these merits, most studies have primarily focused on their linear optical properties under single‐photon excitation. Their potential for nonlinear optical applications, specifically two‐photon absorption (TPA)‐excited emission under near‐infrared (NIR) excitation, remains underexplored and presents significant experimental challenges [11, 12].

TPA‐induced emission is a key enabling mechanism for applications such as deep‐tissue bioimaging, quantum light sources, and infrared communications, as it provides unique advantages such as deeper penetration into scattering media and reduced background signals. However, this inherently nonlinear process typically requires high laser fluences, which can be damaging to biological samples and optical components [13]. Since TPA signals scale with the fourth power of the electric field (E‐field), one effective strategy to enhance TPA‐excited emission is to utilize plasmonic metasurfaces, such as nanohole (NH) arrays [14], bowtie antennas [15], and coaxial apertures [16]. These structures support surface plasmons (SPs) and extraordinary optical transmission (EOT) [14, 17, 18], thereby concentrating the local E‐field and significantly increasing nonlinear optical responses, including second‐harmonic generation (SHG) [19, 20, 21], third‐harmonic generation (THG), and other plasmon‐enhanced nonlinear processes [22, 23, 24].

Over the past decades, a variety of materials have been explored for TPA‐induced emission, including quantum dots (QDs) and organic chromophores. Murray and co‐workers demonstrated that QDs exhibit size‐tunable emission and relatively high quantum yields, but their high surface‐to‐volume ratio can induce severe nonradiative losses [25]. Maruo and co‐workers reported that organic chromophores, despite their facile chemical tunability, typically undergo photochemical degradation under intense excitation [26]. In contrast, metal halide perovskites combine strong optical absorption with large TPA cross‐sections, which makes them highly promising candidates for efficient TPA‐excited photoluminescence (PL) [27]. Despite these advantages, integrating perovskites into plasmonic nanostructures remains a significant challenge due to difficulties in achieving uniform infiltration and in situ crystallization within the confined optical nanocavities.

In this work, we present an integrated approach to address these limitations. We design and fabricate NH arrays to achieve strongly localized E‐field enhancement and high light absorption efficiency under NIR excitation. In addition, we develop a vacuum‐assisted capillary infiltration (VACI) technique that enables uniform delivery and in situ crystallization of bulk‐like CsPbBr_3_ within the NH cavities. To characterize and verify this integration, we introduce a complementary analytical methodology combining a peel‐off process with scanning electron microscopy (SEM), energy‐dispersive x‐ray spectroscopy (EDS), x‐ray diffraction (XRD), and x‐ray photoelectron spectroscopy (XPS), allowing for direct quantitative characterization of the internal filling and interface quality of bulk‐like CsPbBr_3_ within NHs. This approach overcomes the long‐standing challenge of assessing buried interfaces between nanostructures and infiltrated materials, thereby establishing a versatile framework for characterizing future complex nanostructures.

Overall, this research represents a departure from the conventional TPA‐emission platforms, which have predominantly relied on QDs and organic chromophores [25, 26]. By directly integrating bulk‐like perovskites into a plasmonic metasurface, we establish a nonlinear optical platform that enables a highly efficient TPA‐induced emission pathway for next‐generation photonic applications. These include infrared photodetection [28], high‐resolution nonlinear imaging, and quantum light sources [29]. Beyond these applications, our work also introduces a broadly applicable methodology for quantitatively analyzing the infiltration and integration of bulk materials within nanoscale architectures.

## 2 Results and Discussion

### 2.1 Plasmon‐Enhanced TPA‐Excited Emission in Au NHs

Solution‐processable metal halide perovskites have attracted significant interest for next‐generation optoelectronic applications due to their high PLQY and tunable emission wavelengths [30, 31, 32]. In particular, bulk‐like CsPbBr_3_ is attractive as a green‐emitting material, exhibiting high luminescence efficiency and robust optical properties [33]. The tunability of the bandgap via halide composition control allows for precise management of the emission wavelength, positioning perovskites as ideal candidates for nonlinear TPA‐excited PL [34]. This capability is crucial for converting NIR excitation into visible light owing to their favorable bandgap energy and strong TPA response. Furthermore, bulk‐like CsPbBr_3_ films can be fabricated from precursor solutions via simple solution processing, which is advantageous for efficient infiltration and uniform crystal growth within nanostructured cavities [35].

As a critical step toward achieving highly efficient plasmon‐enhanced TPA, we employed three‐dimensional finite‐difference time‐domain (FDTD) simulations using Ansys Lumerical to evaluate the plasmonic enhancement of the Au NH arrays. The simulations focused on predicting the spatial distribution and intensity of the local E‐field enhancement, thereby elucidating the physical mechanism underlying the enhanced TPA‐induced emission efficiency. Subsequently, we fabricated Au NH arrays on glass substrates and analyzed their structural characteristics and plasmonic resonance behavior to experimentally validate the simulation results.

The optical response of the NH array is strongly governed by its structural parameters, including the pitch, hole diameter, and metal film thickness. The pitch primarily determines the grating‐coupling condition for surface plasmon polaritons (SPPs), where increasing the pitch redshifts the resonant wavelength as the momentum‐matching condition shifts toward longer wavelengths [36]. The hole diameter affects both the local field enhancement and the effective refractive index of the metasurface; larger diameters increase aperture coupling, which broadens the resonance peak and induces a redshift due to the modified effective index of the composite layer [37, 38]. The film thickness dictates the strength of evanescent coupling between top and bottom interfaces of the NHs, where thicker films suppress this coupling and reduce overall transmission intensity [39, 40]. Based on these properties, we designed an optimized structure using FDTD simulations to balance transmission, E‐field enhancement, and NH dimensions for effective infiltration and crystallization of bulk‐like CsPbBr_3_.

**FIGURE 1:**
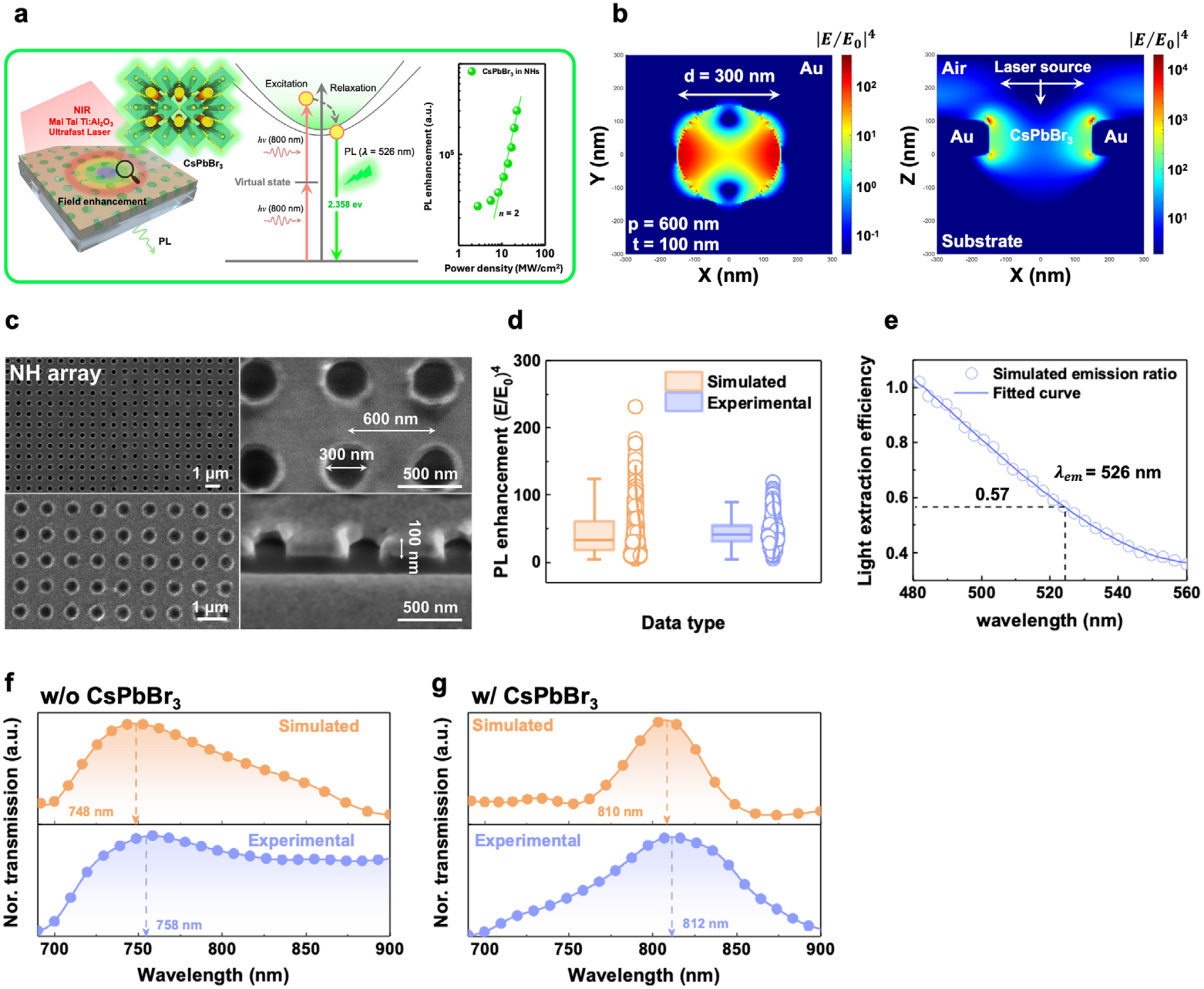
Plasmon‐enhanced TPA‐induced emission in Au NH arrays. (a) Schematic illustration of the plasmon‐enhanced TPA mechanism in bulk‐like CsPbBr_3_ infiltrated into Au NH arrays under 800 nm NIR excitation. The energy diagram highlights the virtual‐state transition and subsequent green PL emission. The inset shows the PL enhancement as a function of peak power density, revealing a quadratic dependence (*n* = 2) consistent with a TPA process. (b) Simulated |E/E_0_|^4^ field enhancement distribution maps of a single NH under 800 nm excitation, showing strong field localization at the hole edges (*d* = 300 nm, *t* = 100 nm, *p* = 600 nm). (c) SEM images of the fabricated Au NH arrays, confirming uniform periodicity and dimensions consistent with simulation parameters. (d) Comparison of simulated and experimental PL enhancement distributions, demonstrating close agreement between theoretical predictions and experimental measurements. (e) Simulated light extraction efficiency of the NH array structure at the CsPbBr_3_ emission wavelength (526 nm), yielding an emission efficiency of ≈57%, which is attributed to partial optical absorption in Au. (f,g) Simulated and experimental normalized transmittance spectra of unfilled and CsPbBr_3_‐filled Au NH arrays. The resonance peak redshifts from 748 nm (simulation) and 758 nm (experiment) for unfilled NHs to 810 nm (simulation) and 812 nm (experiment) after CsPbBr_3_ infiltration, confirming strong agreement between simulation and experiment.

The core concept of this study is illustrated in the schematic presented in Figure 1a. When a NIR excitation laser is incident on the Au NH array, SPs are excited and concentrated into localized E‐field hotspots within the NHs. These localized E‐fields play a crucial role in increasing the TPA efficiency of bulk‐like CsPbBr_3_ infiltrated within the NH cavities. This is because TPA is a nonlinear process that scales with the square of the excitation intensity and thus the fourth power of the E‐field [41, 42]. Notably, the photon energy of 800 nm excitation (1.55 eV) is sufficient to drive TPA, as two combined photons (3.10 eV) exceed the direct bandgap of bulk‐like CsPbBr_3_ (≈2.36 eV, Figure S1), demonstrating its suitability for NIR‐driven TPA‐induced emission. Consequently, the enhanced TPA results in a significant increase in green PL emission, as described by the energy diagram on the right side of Figure 1a.

To thoroughly understand the enhancement mechanism originating from the Au NH array, the results of FDTD simulations are depicted in Figure 1b. The simulation model reflected the experimental environment, assuming uniform infiltration and crystallization of bulk‐like CsPbBr_3_ within the NH cavities on a glass substrate. However, in the fabricated samples, surface roughness near the edges of the NHs was present due to perovskite crystallization, which was not modeled. The simulations revealed the formation of strongly localized hotspots and suggested that the average E‐field amplitude within the NHs is enhanced by approximately threefold. Furthermore, unlike many plasmonic nanostructures where a dielectric spacer layer is introduced between the metal and the emitter to suppress PL quenching by blocking non‐radiative electron‐hole recombination at the metallic interface, such a spacer layer was intentionally omitted in this study. Owing to the bulk‐like morphology of CsPbBr_3_, the contact area between the CsPbBr_3_ and the Au surface is inherently limited relative to the total volume, thereby reducing non‐radiative interfacial recombination without the need for an additional dielectric interlayer.

To validate the structural parameters used in the simulation (*d* = 300 nm, *t* = 100 nm, *p* = 600 nm), further characterization was performed. Atomic force microscopy (AFM) analysis confirmed crystal grain sizes of ≈100 to 200 nm (Figure S2), sufficiently small to allow effective filling of 300 nm‐diameter NHs. Additionally, SEM images confirmed that the fabricated Au NHs exhibited well‐defined periodicity and dimensions consistent with the simulation parameters (Figure 1c).

To quantitatively assess the plasmon‐enhanced TPA efficiency, we compared the simulated E‐field enhancements with experimentally observed PL intensity enhancements (Figure 1d). The simulations predicted an average |*E/E_0_* |^4^ PL enhancement of ≈75.52 within the NHs. However, the simulated light extraction efficiency at the CsPbBr_3_ emission wavelength (526 nm) was approximately 57% (Figure 1e), primarily due to parasitic absorption by Au, which reduces the effective extractable PL. Accounting for this extraction efficiency, the net theoretical PL enhancement is approximately 43. This value matches quite well with the experimentally measured PL enhancement of ≈40.33. A comparison of TPA/PL enhancement factors reported for representative plasmonic and photonic nanostructures is provided in Table S1. The small discrepancy between the theoretical and experimental values is primarily attributed to practical factors such as sample quality (surface defects and roughness) and optical system losses during measurement [43, 44, 45, 46]. Nevertheless, this remarkable agreement between the simulated prediction and the experimental measurement strongly demonstrates that the plasmon resonance induced by the Au NH array effectively enhances the TPA‐excited emission efficiency of bulk‐like CsPbBr_3_.

Finally, the normalized transmittance spectra of the Au NH array were compared with FDTD simulations (Figure 1f,g). For the unfilled NH array, resonance peaks were observed at 748 nm (simulation) and 758 nm (experiment). Upon CsPbBr_3_ infiltration, the resonance redshifted to 810 nm (simulation) and 812 nm (experiment), consistent with the increased effective refractive index inside the NHs. This excellent agreement verifies that our simulation model accurately predicts the plasmonic resonance behavior of the NH arrays and serves as an important basis for validating the reliability of the E‐field and PL enhancement analyses presented above.

These results clearly indicate that plasmonic engineering is a powerful strategy for controlling and amplifying nonlinear optical processes, highlighting its potential for the development of highly efficient TPA‐excited emission‐based applications.

### 2.2 Uniform Infiltration and In Situ Crystallization of CsPbBr_3_ Within NHs

Conventional fabrication methods, such as the spin‐coating process, often result in poor infiltration of solution into nanometer‐sized features, thereby failing to achieve selective growth and uniform filling of plasmonic NH arrays. This leads to sporadic crystal growth on the surface rather than within the confined nanostructures, which compromises the plasmon‐exciton coupling and ultimately degrades overall optical performance [47, 48]. To overcome this critical limitation, we have developed a novel and simple approach using VACI, which enables precise and uniform selective growth of bulk‐like CsPbBr_3_ within the NH arrays.

**FIGURE 2:**
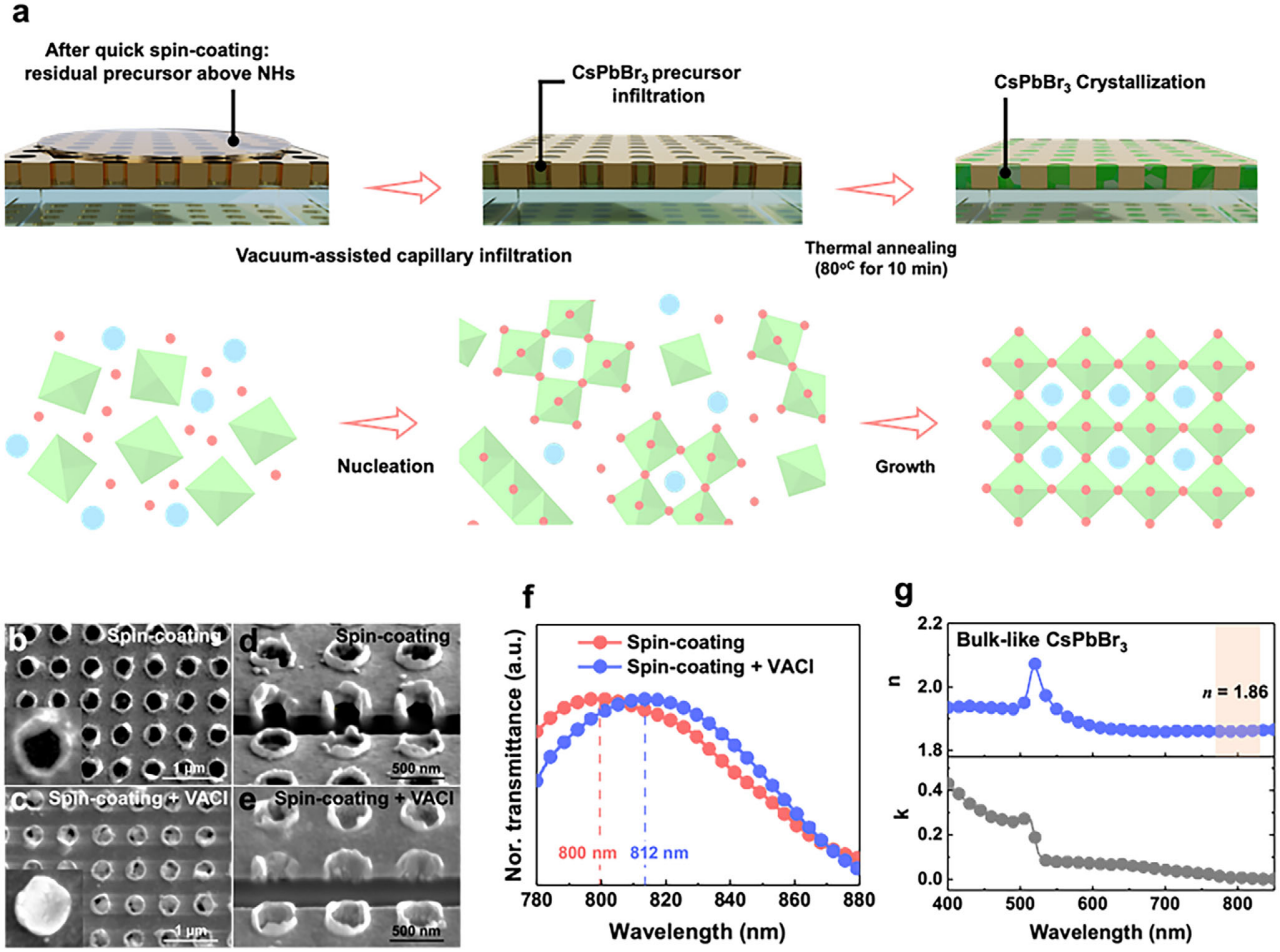
Uniform infiltration and in situ crystallization of bulk‐like CsPbBr_3_ within NHs via VACI. (a) Schematic illustration of the VACI mechanism: a rapid spin‐coating step leaves a residual precursor layer above the NHs, followed by vacuum‐assisted capillary infiltration and subsequent in situ crystallization under thermal annealing at 80°C for 10 min. (b–e) Top‐view and cross‐sectional SEM images comparing samples fabricated with conventional spin‐coating (b,d) and with spin‐coating combined with VACI (c,e). Magnified views of individual NHs highlight the uniform and significantly improved cavity filling achieved by the VACI process. (f) Normalized transmittance spectra of NH arrays fabricated with and without VACI. A distinct redshift of ≈12 nm (from 800 to 812 nm) is observed after VACI, attributed to the increased effective refractive index inside the NHs upon uniform filling of CsPbBr_3_. (g) Experimentally measured refractive index (*n*) and extinction coefficient (*k*) spectra of bulk‐like CsPbBr_3_ obtained by spectroscopic ellipsometry, confirming *n* = 1.86 at 800 nm.

The mechanism of this process is schematically illustrated in Figure 2a. The process begins with a rapid spin‐coating step, which produces a thin layer of residual CsPbBr_3_ precursor solution on the surface of the NH arrays. This step is crucial as it drives the constituent ions (Cs^+^, Br^−^, Pb^2+^) into close proximity, thereby facilitating the subsequent crystallization. The sample is then immediately transferred to a vacuum chamber. Due to the hydrophilic nature of the NHs rendered by O_2_ plasma treatment, the VACI mechanism drives the residual precursor solution deep into the NH interiors through capillary action under reduced pressure. This infiltration is highly efficient, as the small, uniform size of the synthesized bulk‐like CsPbBr_3_ (≈100 to 200 nm in diameter, as confirmed by Figure S2) is well‐suited for effective filling of the NH arrays. Following infiltration, the sample is rapidly transferred to a hotplate for thermal annealing at 80°C for 10 min. This thermal treatment supplies the energy required to surpass the nucleation barrier (ΔG^*^), initiating the in situ crystallization of bulk‐like CsPbBr_3_ within the confined spaces of the NH arrays [49, 50].

To evaluate the efficiency of the VACI method, we performed a comparative analysis using SEM to contrast films fabricated with and without VACI. As shown in the top‐view (Figure 2b,c) and cross‐sectional images (Figure 2d,e), the film prepared with conventional spin‐coating exhibits poor infiltration, with non‐uniformly filled holes and a majority of the crystallization occurring on the surface rather than deep within the nanostructures. In contrast, films fabricated using VACI demonstrate effective infiltration. The top‐view SEM image reveals a dense and uniform distribution of bulk‐like CsPbBr_3_, while the cross‐sectional view confirms tight filling throughout the NH depth with high‐quality, uniformly crystallized material. This qualitative observation was further supported by a statistical filling‐fraction analysis (Figure S3), which showed that the VACI‐processed sample contained 25% fully filled, 40% partially filled, and 35% empty NHs, whereas the spin‐coated sample contained only 0.3% fully filled, 23% partially filled, and 77% empty cavities. These combined morphological and statistical results confirm that the VACI method enables significantly more uniform infiltration and in situ crystallization of bulk‐like CsPbBr_3_ within the plasmonic NH arrays.

Furthermore, we investigated the effect of VACI by comparing the normalized transmittance spectra of NH arrays fabricated with and without VACI. The objective of this test was to spectrally verify the presence of CsPbBr_3_ within the NHs. Figure 2f shows that the spectrum of the spin‐coated sample exhibits a resonance peak at 800 nm, while the VACI‐treated sample shows a distinct redshift to 812 nm. This ≈12 nm shift can be attributed to the increased effective refractive index inside the NH cavities (*n* = 1.86 at around 800 nm for bulk‐like CsPbBr_3_, Figure 2g) upon uniform filling. This shift was also confirmed from electromagnetic simulations, showing excellent agreement with the experimental results (Figure S4). The simulations also revealed a slight decrease in transmittance intensity, which is attributed to the higher refractive index of CsPbBr_3_ (*n* ≈ 1.86) compared to air (*n* ≈ 1.0), leading to stronger interfacial reflection at the Au‐CsPbBr_3_‐glass boundaries [51]. Such a spectral shift strongly confirms the successful infiltration and crystallization of bulk‐like CsPbBr_3_ within the NH interiors, providing independent optical evidence that complements the morphological analyses. Overall, the complementary structural and optical characterizations clearly validate the effectiveness of the VACI method, establishing it as a robust strategy for selective and uniform integration of bulk‐like perovskites into nanostructured plasmonic platforms.

### 2.3 Direct Visualization of Infiltration and In Situ Crystallization via Peel‐Off Method

Conventional top‐view SEM is limited in verifying the infiltration depth of nanostructures. To directly assess the filling uniformity of bulk‐like CsPbBr_3_ within the entire depth of the NH arrays, we developed a peel‐off methodology for depth‐resolved morphological and chemical analysis.

**FIGURE 3:**
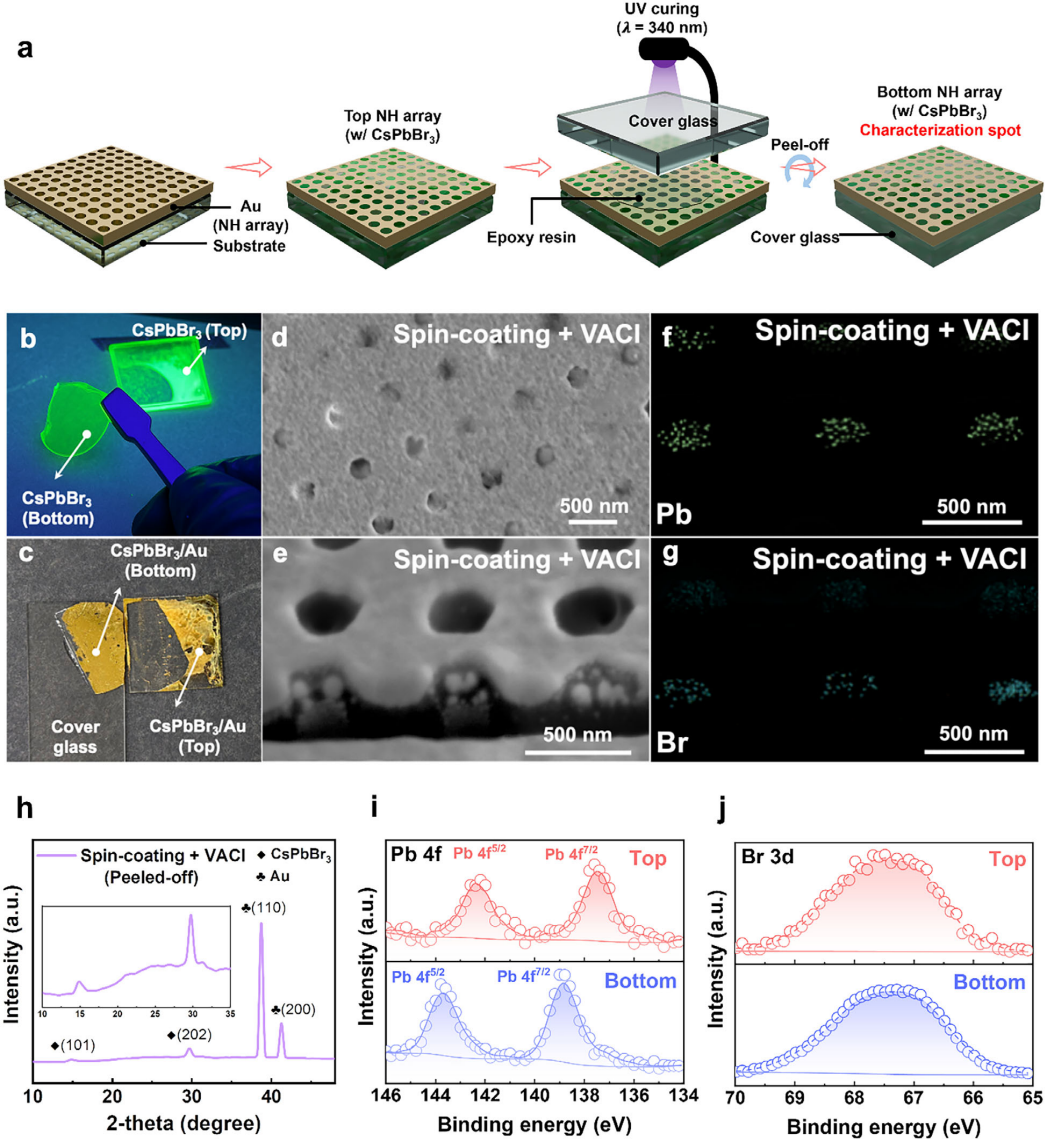
In‐depth morphological and chemical analysis of bulk‐like CsPbBr_3_ infiltration within Au NHs using a peel‐off technique. (a) Schematic illustration of the peel‐off procedure: after CsPbBr_3_ infiltration via VACI, an epoxy resin layer and cover glass were applied, followed by UV curing and mechanical separation, enabling direct access to the underside of the NH array. (b) Photographs of the transferred CsPbBr_3_ film on glass, showing preserved green fluorescence (*λ* ≈ 526 nm). (c) Photograph of the remaining Au NH array after peel‐off, confirming structural integrity. (d,e) SEM images of peeled‐off samples prepared with VACI, revealing uniform infiltration and dense crystallization of CsPbBr_3_ through most of the NH cavity depth. (f,g) Corresponding EDS elemental mapping confirms the presence of Pb and Br signals from the deep regions of the NHs. (h) XRD patterns of peeled‐off films prepared with VACI, showing distinct CsPbBr_3_ diffraction peaks at 15.3° and 30.6° ((101) and (202) planes), indicative of the orthorhombic bulk‐like phase. (i,j) XPS spectra of Pb 4f and Br 3d signals observed from both top and bottom regions of the peeled‐off samples.

The entire peel‐off process is schematically illustrated in Figure 3a. After efficiently infiltrating the NH arrays with bulk‐like CsPbBr_3_ using the VACI method, an epoxy resin was drop‐cast onto the surface, covered with glass, and cured under ultraviolet (UV) light (*λ* = 340 nm). Following polymerization, the NH array was peeled away from the sample and attached to the glass substrate. This procedure exposes the underside of the nanostructures, enabling direct visualization of the infiltration depth. Figure 3b reveals that the bulk‐like CsPbBr_3_ film was successfully transferred onto the glass substrate with minimal damage, while retaining its characteristic green fluorescence (λ ≈ 526 nm). Figure 3c further confirms that the Au NH array remained structurally intact after the peel‐off process, indicating that the mechanical separation did not compromise the plasmonic framework. Complementary transmittance spectra (Figure S5) exhibited negligible spectral shifts after peel‐off, demonstrating that the structural and optical integrity of the NH arrays was well‐preserved.

To further evaluate the infiltration quality achieved by the VACI method, we examined peeled‐off samples by SEM and EDS. The top‐view SEM (Figure 3d) reveals a clean and uniform distribution of bulk‐like CsPbBr_3_ within the deep regions of the NH arrays. The cross‐sectional SEM (Figure 3e) further confirms dense and nearly continuous filling throughout most of the cavity depth, indicating successful in situ crystallization within the confined nanostructures. The strong brightness of CsPbBr_3_ in the cross‐section is consistent with the higher atomic number of Pb (*Z* = 82) relative to Au (*Z* = 79), which enhances electron backscatter [52]. Complementary EDS analysis verified the chemical composition, showing clear Pb (Figure 3f) and Br (Figure 3g) signals from the deep‐level regions, providing strong evidence of substantially increased infiltration and crystallization within the NH interiors.

The crystallinity of the infiltrated bulk‐like CsPbBr_3_ was further studied by XRD analysis of the peeled‐off samples (Figure 3h). Distinct diffraction peaks were observed at 15.3° and 30.6°, corresponding to the (101) and (202) planes of bulk‐like CsPbBr_3,_ indicative of its orthorhombic crystal phase [33], consistent with the reference CsPbBr_3_ film on glass (Figure S6). Additional peaks at ∼38° and ∼41° arise from the underlying Au NH arrays [53], confirming that the diffraction signals originate from perovskite domains embedded within the NHs rather than surface residues.

XPS analysis provided complementary chemical evidence of infiltration across different depths. The Pb 4f spectra (Figure 3i) collected from both the top and bottom regions of the peeled‐off sample exhibited a slight binding energy shift compared to the reference CsPbBr_3_ film on glass (Figure S7), which might be attributed to electronic density redistribution at the Au‐CsPbBr_3_ interface [54]. In contrast, the Br 3d spectra (Figure 3j) showed a negligible shift, indicating that the bromide environment remained unchanged. Consequently, these results provide strong structural and chemical evidence that bulk‐like CsPbBr_3_ was effectively infiltrated and crystallized within the confined NH arrays.

### 2.4 Plasmonically Enhanced Optical Properties of CsPbBr_3_ in NH Arrays

**FIGURE 4:**
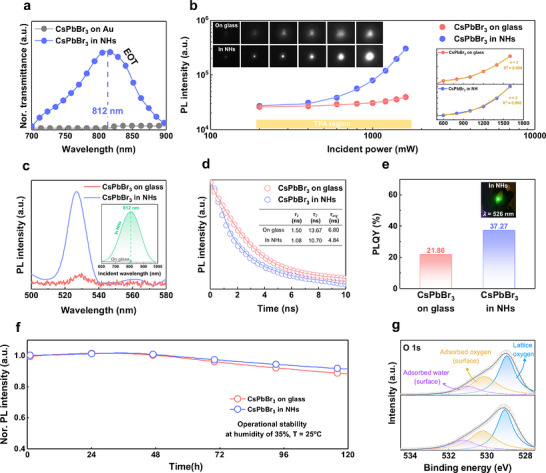
Plasmonically enhanced TPA‐excited emission and long‐term operational stability of bulk‐like CsPbBr_3_ in Au NHs. (a) Normalized transmittance spectra of bulk‐like CsPbBr_3_ on an unpatterned Au film and within Au NH arrays, illustrating the extraordinary optical transmission through the CsPbBr_3_‐filled NHs, with the resonance at 812 nm. (b) Log‐log plot of TPA‐excited PL intensity as a function of incident NIR excitation power (mW), demonstrating the characteristic quadratic relationship consistent with a TPA mechanism. Insets show the corresponding TPA‐excited emission images and fitted curves. (c) TPA‐excited PL spectra of bulk‐like CsPbBr_3_ on glass and in NHs under 800 nm NIR excitation, showing enhanced emission intensity in NHs. The inset presents the TPA‐excited PL intensity as a function of incident wavelength. (d) TRPL decay curves and corresponding lifetime values measured under UV excitation (340 nm). (e) Comparison of PLQY for bulk‐like CsPbBr_3_ on glass and in NHs under UV excitation (340 nm). The inset shows a photograph of the green emission from the CsPbBr_3_ in NHs. (f) Long‐term operational stability of the NIR‐driven PL intensity of both bulk‐like CsPbBr_3_ on glass and in NHs, measured at 35% relative humidity and 25°C. (g) XPS analysis of the O 1s core level for bulk‐like CsPbBr_3_ in NHs before and after 5 days, showing minimal changes, which suggests negligible environmental degradation.

To comprehensively elucidate the impact of the plasmonic NH arrays on the optical properties and stability of CsPbBr_3_ films, further characterization was performed. Figure 4a illustrates the EOT characteristics of the film incorporating NHs. The transmittance spectra of the bulk‐like CsPbBr_3_ in NHs show enhanced transmittance compared to CsPbBr_3_ on the unpatterned Au film at the targeted NIR wavelength. The weak transmission feature observed in the CsPbBr_3_/Au can be attributed to the opacity of the continuous Au film and mild thin‐film interference between air/CsPbBr_3_ and CsPbBr_3_/Au interfaces [55]. Specifically, a notable increase in transmittance is observed around the excitation wavelength of (800 nm), which is crucial for maximizing the efficiency of NIR‐based nonlinear processes. The periodic arrangement of NHs allows for efficient coupling of light to SP modes within the NH arrays [17, 56]. These excited SP modes generate enhanced electromagnetic fields within the CsPbBr_3_‐filled cavities, thereby increasing the local photon density and enhancing the probability of TPA events.

The core demonstration of the enhanced nonlinear optical properties is depicted in Figure 4b, which plots the fluorescence intensity as a function of incident power for the bulk‐like CsPbBr_3_ in NHs and on glass films under NIR excitation. Both samples exhibit a distinct nonlinear increase in fluorescence intensity with increasing incident power, a characteristic of a TPA process [57]. Importantly, the bulk‐like CsPbBr_3_ in NHs exhibits a substantially higher fluorescence intensity compared to the bulk‐like CsPbBr_3_ on glass. Furthermore, the bulk‐like CsPbBr_3_ in NHs shows a faster increase in nonlinear emission at lower excitation powers. This pronounced enhancement of nonlinear emission in the presence of NH arrays highlights their critical role in amplifying the TPA efficiency. The inset images visually confirm that the emission area of the bulk‐like CsPbBr_3_ in NHs is significantly brighter as the incident power increases, highlighting the practical advantage of this architecture for TPA‐excited emission‐based applications.

Figure 4c provides spectral evidence of the green emission from bulk‐like CsPbBr_3_ within NHs when excited by NIR light through the TPA process. The inset presents the TPA‐excited PL intensity as a function of incident wavelength for CsPbBr_3_ in NHs and on glass, confirming that the emission enhancement is maximized near 800 nm, consistent with the peak laser intensity of the Ti:Sapphire laser and the broad plasmonic resonance of the CsPbBr_3_‐filled NH arrays. Compared to the PL spectrum under UV excitation (Figure S8), the emission peak exhibits a slight redshift of ≈5 nm. This shift is attributed to bandgap shrinkage effects induced by high carrier densities generated during the TPA process, where the simultaneous absorption of two photons leads to a transient carrier accumulation in the conduction band, narrowing the effective bandgap and producing a redshifted emission [58].

To elucidate the underlying mechanism for the observed emission enhancement, time‐resolved photoluminescence (TRPL) measurements were performed under UV excitation (Figure 4d), revealing a faster decay for bulk‐like CsPbBr_3_ in NHs compared with CsPbBr_3_ on glass. While this shorter lifetime could be attributed to defect‐induced non‐radiative loss [59], this possibility is considered unlikely based on the PLQY measurements presented in Figure 4e. The PLQY increases from ≈22% (CsPbBr_3_ on glass) to 37% (CsPbBr_3_ in NHs), suggesting that defects are not the primary cause of the faster decay. From the PLQY and TRPL results, the Purcell factor can be estimated as $F_{p}=\frac{\tau_{g}}{\tau_{NH}}$, where *F_p_* is the Purcell enhancement factor, τ_*g*_ is the PL decay lifetime of bulk‐like CsPbBr_3_ without the NH array, and τ_*NH*_ is the corresponding lifetime of CsPbBr_3_ within the NH array. This yields a calculated Purcell factor of ≈1.4, suggesting a modest enhancement of the radiative emission rate at the emission wavelength under one‐photon excitation. This enhancement is potentially associated with the modification of the local density of optical states (LDOS) near the CsPbBr_3_ emission wavelength, induced by the plasmonic resonance of the Au NH arrays [60, 61]. Whether this enhancement persists under TPA excitation remains an open question and warrants further investigation.

The long‐term operational stability of NH‐incorporated bulk‐like CsPbBr_3_ is a critical factor for practical applications. Figure 4f illustrates the remarkable stability of the TPA‐based luminescence from the bulk‐like CsPbBr_3_ in NHs. The fluorescence intensity remains highly stable over five days. Interestingly, a slight increase in luminescence intensity is observed during the initial hours, prior to reaching a stable state, which might be attributed to a self‐healing mechanism induced by the low‐energy NIR irradiation [60]. This NIR‐driven two‐photon‐excited emission exhibits significantly superior stability compared to conventional UV‐excited systems, which typically undergo rapid degradation due to their poor environmental stability and the high energy of the incident photons.

To further examine the contributing factors to this exceptional stability, we performed XPS analysis of the O 1s core level (Figure 4g). The O 1s spectra reveal three distinct peaks corresponding to adsorbed water (531.5 eV), adsorbed oxygen (530.2 eV), and lattice oxygen (528.8 eV) [61]. The minimal increase in adsorbed oxygen and water, as well as lattice oxygen peaks, even after 5 days, suggests that surface degradation from environmental exposure is negligible. This is likely due to the physical confinement provided by the NH structure, which minimizes the exposed surface area of the bulk‐like CsPbBr_3_ to the environment. This is complemented by the photothermal analysis, as shown in Figure S9, which exhibits a minimal temperature increase from 26.78°C to 28.81°C under continuous 800 nm NIR laser illumination. This negligible temperature rise indicates that photothermal heating does not cause significant degradation, further supporting the robust nature of this TPA‐driven emission system.

## 3 Conclusion

In conclusion, we have developed a VACI fabrication strategy that enables the uniform delivery and in situ crystallization of bulk‐like CsPbBr_3_ within plasmonic NH metasurfaces. This integrated fabrication and characterization methodology represents a significant advance in overcoming long‐standing challenges associated with efficient material infiltration and direct evaluation of buried interfaces in complex nanostructures. By combining VACI with a complementary peel‐off‐based analytical approach to probe the underside of the NH array, we establish a versatile framework for quantitatively probing material‐structure integration at the nanoscale.

This study demonstrates a nonlinear optical platform that departs from conventional TPA‐based systems based on quantum dots and organic chromophores. The localized electromagnetic field amplification provided by the plasmonic NH arrays significantly enhances TPA efficiency even under comparatively low excitation fluences, which minimizes potential sample degradation while maximizing the nonlinear response. Additionally, the plasmonic resonance of the Au NH arrays modifies the surrounding photonic environment near the CsPbBr_3_ emission wavelength, which is responsible for the modest Purcell enhancement.

These findings not only offer a pathway toward high‐efficiency infrared photodetection, high‐resolution nonlinear imaging, and quantum light sources, but also introduce a broadly applicable methodology for integrating and characterizing bulk functional materials in plasmonic and nanophotonic architectures.

## 4 Experimental Section

### 4.1 Materials

Lead(II) bromide (PbBr_2_, 99.999%), cesium bromide (CsBr, 99%), and dimethyl sulfoxide (DMSO, 99.9%) were purchased from Sigma‐Aldrich and used directly without additional purification unless specified otherwise.

### 4.2 Perovskite Precursor Solutions

Precursor solutions were prepared by dissolving CsBr and PbBr_2_ in anhydrous DMSO at a Cs:Pb molar ratio of ∼1.7. To facilitate capillary infiltration into nanostructures, the solutions were slightly diluted prior to use. The mixtures were stirred overnight at 60°C and filtered through 0.45 µm polytetrafluoroethylene syringe filters before application.

### 4.3 Device Fabrication

First, glass substrates were sequentially cleaned with acetone, methanol, and isopropanol, followed by a dehydration bake at 200°C for 10 min. A 2.5 nm Ti adhesion layer and a 100 nm Au film were deposited by sputtering (AJA2, Minnesota Nanocenter). The Ti adhesion layer was excluded from the peel‐off sample. The NH arrays were patterned by electron beam lithography (Vistec EBPG5000+) using 950 poly(methyl methacrylate) (PMMA) C4 resist (4000 rpm, 60 s; 180°C, 2 min; exposure dose 1000 µC/cm^2^), developed in methyl isobutyl ketone (MIBK):isopropanol (1:3) for 50 s, and then transferred into the Au layer by ion milling (Intivac, Ar plasma). After etching, the residual resist was removed by acetone and O_2_ plasma cleaning.

Prior to perovskite deposition, the nanohole arrays were treated with O_2_ plasma to render the surface hydrophilic. The synthesized CsPbBr_3_ precursor solution was then spin‐coated onto the arrays at 4000 rpm for 20 s and immediately subjected to VACI under reduced pressure to fill the NH cavities. The films were subsequently transferred to a hot plate and annealed at 80°C for 10 min.

### 4.4 Optical Simulation

Numerical simulations were carried out using Ansys Lumerical FDTD. A 3D unit cell containing a NH at the center of an Au film was constructed, with the lateral dimensions equal to the array period. A linearly polarized plane wave was injected along the *z*‐axis from air into the glass substrate. Anti‐symmetric and symmetric boundary conditions were applied along the x‐ and y‐axes, respectively, to reproduce the periodic structure, while perfectly matched layers were used along the *z*‐axis. The simulation region was set to 11 µm in the *z*‐direction based on convergence testing. Transmission spectra were collected from a monitor plane placed 1.5 µm below the NH array. Electric field distributions were obtained using frequency‐domain monitors in the (x,y) and (x,z) planes. Optical constants for Au and SiO_2_ were built‐in Ansys Lumerical values obtained from Ref. [62], and the CsPbBr_3_ layer was modeled with a constant refractive index of *n* = 1.86.

The simulation of the light extraction efficiency was also performed in Ansys Lumerical FDTD. The same conditions were followed for the NH transmission and PL enhancement simulations, but the source was changed to a dipole placed within the perovskite layer. The transmission was measured at a monitor plane placed 1.5 µm below the surface of the perovskite layer. For the emission efficiency of the perovskite thin film, the gold layer was disabled, and the transmission spectra were collected.

### 4.5 Characterization

The morphology of the samples was examined using a high‐resolution field‐emission scanning electron microscope (FE‐SEM, Thermo Fisher Helios 5 DualBeam). Structural analysis was carried out using an x‐ray diffractometer (XRD, Rigaku SmartLab XE). The optical transmittance was measured using a Nikon Eclipse Ti‐S microscope equipped with an Ocean Optics QE65000 spectrometer. The steady‐state photoluminescence (PL) spectra (*λ_ex_* ≈ 800 nm, two‐photon absorption) were measured by Ocean Optics Flame Spectrometer, while the photoluminescence quantum yield (PLQY) and time‐resolved photoluminescence (TRPL) decay (*λ_ex_* ≈ 340 nm) were characterized using a spectrofluorometer (FS5, Edinburgh). Surface topography was investigated by atomic force microscopy (AFM, CoreAFM, Nanosurf). The chemical composition and electronic states were analyzed by x‐ray photoelectron spectroscopy (XPS, PHI 5000 VersaProbe III). The complex refractive index (*n*, *k*) of the CsPbBr_3_ films was determined by spectroscopic ellipsometry (VASE, J.A. Woollam) over the wavelength range of 350–900 nm, and fitted using a multi‐layer optical model to extract optical constants.

The incident‐power‐dependent PL intensity and long‐term stability were evaluated under excitation by a Mai Tai Ti:sapphire femtosecond laser (*λ_ex_* ≈ 800 nm, two‐photon absorption) under dark conditions. Fluorescence intensity was measured from the backside of the sample using an Allied Vision Prosilica GT 1920 charge‐coupled device (CCD) camera. A 750 nm short pass filter was added to the optical path to remove any potential background intensity from the incident laser. The average power of the laser was measured from a partially reflecting mirror using a Thorlabs Power Meter. All measurements of the films were conducted under ambient air conditions (*T* ≈ 25°C, relative humidity ≈ 35%) without any encapsulation process.

## Conflicts of Interest

The authors declare no conflicts of interest.

## Supporting information




**Supporting File**: adma73835‐sup‐0001‐SuppMat.docx.

## Data Availability

The data that support the findings of this study are available from the corresponding author upon reasonable request.
