# Supplementary material for: Selective Growth of Bulk‐Like Perovskite in Plasmonic Nanoholes for Enhanced Two‐Photon‐Excited Emission
Source: Adv Mater. 2026 Jul 9;38(45):e73835. doi: 10.1002/adma.73835 (PMC13471766; doi:10.1002/adma.73835)
Supplement: Supplementary file 1 [file ADMA-38-e73835-s001.docx]

*Supporting Information*

**Selective Growth of Bulk-Like Perovskite in Plasmonic Nanoholes for Enhanced Two-Photon-Excited Emission**

*Jung-Jae Do*^a,b^*, Bryan K Chantigian*^b^*,* *Daniel Upcraft*^b^*, Wonwoo Lee*^b^*, Ho-Sung Shin^a^, Nathan C. Lindquist*^c^*, Jae Woong Jung*^d,e^*, Bharat Jalan^a^,* *and Sang-Hyun Oh*^b,*^

^a^Department of Chemical Engineering and Materials Science, University of Minnesota, Minneapolis, Minnesota 55455, United States

^b^Department of Electrical and Computer Engineering, University of Minnesota, Minneapolis, Minnesota 55455, United States

^c^Department of Physics and Engineering, Bethel University, St Paul, Minnesota 55112, United States

^d^Department of Materials Science and Engineering, College of Engineering, Kyung Hee University, 1732 Deogyeong-daero, Giheung-gu, Yongin-si, Gyeonggi-do 446-701, Republic of Korea

^e^Integrated Education Institute for Frontier Materials (BK21 Four), Kyung Hee University, 1732 Deogyeong-daero, Giheung-gu, Yongin-si, Gyeonggi-do 446-701, Republic of Korea

| **Structure** | **Material** | **TPA/PL enhancement** | **Wavelength** | **Reference** |
| --- | --- | --- | --- | --- |
| CuS nanodisk | CuS | 3× | 855 nm | 63 |
| Si photonic crystal | CsPbBr_3_ QD | 15× | 925 nm | 64 |
| Au nanorod & nanocluster | Au nanoclusters | 25× | 850 nm | 65 |
| Au nanohole | Bulk CsPbBr_3_ | 40.33× | 800 nm | This work |
| Au nanoantenna | Organic molecule | 53× | 600 nm | 66 |

**Table S1.** Comparison of TPA/PL enhancement factors reported for representative plasmonic and photonic nanostructures.

**Figure S1.** Tauc plot of the bulk-like CsPbBr₃ film on a glass substrate, revealing a direct optical bandgap of ≈2.36 eV. This value is well-suited for NIR-driven TPA-excited emission, as two 800 nm photons (3.10 eV) exceed the bandgap energy.

**Figure S2.** AFM image of the bulk-like CsPbBr₃ film, showing that the majority of grains fall within 100 to 200 nm, which is compatible with the 300 nm diameter of the NH arrays and facilitates efficient infiltration and crystallization.

**Figure S3.** Filling fraction analysis of NH arrays fabricated with conventional spin-coating and by spin-coating combined with VACI. The VACI-treated sample shows a markedly higher filling efficiency.

**Figure S4.** Simulated transmittance spectra of Au NH arrays before and after CsPbBr₃ infiltration, showing a redshift (≈9 nm) and slight attenuation due to the increased refractive index (*n* ≈ 1.86).

**Figure S5**. Normalized transmittance spectra comparing the as-fabricated Au NH array with its peeled-off counterpart. The close agreement of resonance peaks indicates that the peel-off procedure preserves the intrinsic optical properties of the nanostructures.

**Figure S6.** XRD pattern of a bulk-like CsPbBr₃ film on glass, exhibiting characteristic diffraction peaks at 15.3°, 21.6°, and 30.6°, corresponding to the (101), (121), and (202) planes of orthorhombic CsPbBr₃, respectively. These results confirm the orthorhombic crystalline phase of the synthesized CsPbBr₃.

**Figure S7.** High-resolution XPS spectra of Pb 4f and Br 3d for a CsPbBr₃ film on glass, serving as a reference for evaluating the chemical integrity of CsPbBr₃ in peeled-off samples.

**Figure S8.** Absorption and photoluminescence (PL) spectra of a CsPbBr₃ film under UV excitation (*λ* = 340 nm), showing a strong green emission peak centered at 521 nm (inset: photograph of the luminescence).

**Figure S9.** Minimal photothermal heating of the bulk-like CsPbBr₃ within NHs under continuous 800 nm NIR illumination. Thermal images recorded every 1 h show only a slight temperature increase from ~26.78°C to ~28.81°C over 3 h, indicating that photothermal heating does not contribute to degradation.

**References**

63. B. C. Marin, S. W. Hsu, L. Chen, et al., "Plasmon-Enhanced Two-Photon Absorption in Photoluminescent Semiconductor Nanocrystals," ACS Photonics 3 (2016): 526–531, <https://doi.org/10.1021/acsphotonics.6b00037>.

64. C. Becker, S. Burger, C. Barth, et al., "Nanophotonic-Enhanced Two-Photon-Excited Photoluminescence of Perovskite Quantum Dots," ACS Photonics 5 (2018): 4668–4676, <https://doi.org/10.1021/acsphotonics.8b01199>.

65. A. Pniakowska and J. Olesiak-Banska, "Plasmonic Enhancement of Two-Photon Excited Luminescence of Gold Nanoclusters," Molecules 27 (2022): 807, <https://doi.org/10.3390/molecules27030807>.

66. V. P. Drachev, A. V. Kildishev, J. D. Borneman, et al., "Engineered Nonlinear Materials Using Gold Nanoantenna Array," Scientific Reports 8 (2018): 780, <https://doi.org/10.1038/s41598-017-19066-3>.
